# Durable proteo-hybrid vesicles for the extended functional lifetime of membrane proteins in bionanotechnology†
†Electronic supplementary information (ESI) available: Additional supporting data and experimental methods. See DOI: 10.1039/c6cc04207d



**DOI:** 10.1039/c6cc04207d

**Published:** 2016-08-16

**Authors:** Sanobar Khan, Mengqiu Li, Stephen P. Muench, Lars J. C. Jeuken, Paul A. Beales

**Affiliations:** a School of Chemistry and Astbury Centre for Structural Molecular Biology , University of Leeds , Leeds , LS2 9JT , UK . Email: p.a.beales@leeds.ac.uk; b School of Biomedical Sciences and Astbury Centre for Structural Molecular Biology , University of Leeds , Leeds , LS2 9JT , UK

## Abstract

The full capabilities of membrane proteins in bionanotechnology can only be realised through improvements in their reconstitution environments that combine biocompatibility to support function and durability for long term stability. We demonstrate that hybrid vesicles composed of natural phospholipids and synthetic diblock copolymers have the potential to achieve these criteria.

## Abstract 2

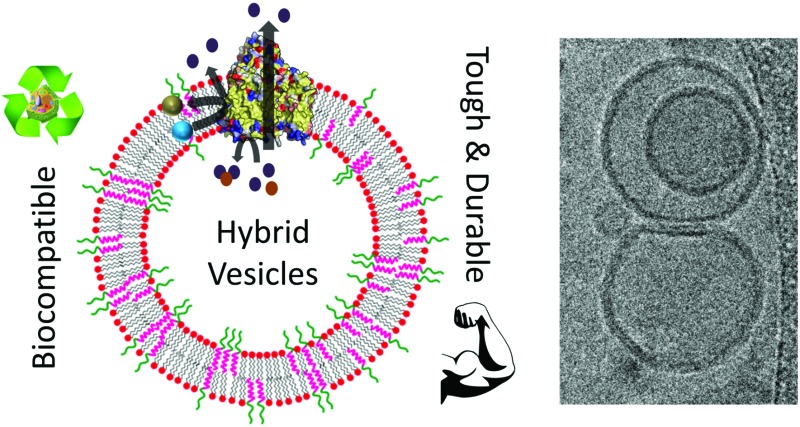
Significant enhancement of membrane protein functional durability is demonstrated when reconstituted in hybrid lipid–block copolymer vesicles compared to conventional proteoliposomes.

Membrane proteins have great potential for applications in nascent bionanotechnologies.^1^ This large class of proteins, an estimated 30% of proteins encoded by eukaryotic genomes, consists of a wide variety of ion pumps, transporters, motors, signal transducers, catalysts and adhesion molecules. Such highly sophisticated macromolecules could play important functional roles within the design of biosensors, drug delivery systems, nanoreactors, energy capture and storage devices, or artificial cells. A key consideration when designing these advanced materials is their functional lifetime; liposomes may not always be the ideal reconstitution system due to their relatively poor long term stability when compared to synthetic alternatives, such as polymersomes.^2^ However, the natural biocompatibility of the lipid bilayer structure and, in some cases, specific lipid–protein interactions are often a requirement for membrane protein function.^3^ Here, we address this challenge by developing proteo-hybrid vesicles (PHVs) as reconstitution systems that combine biocompatibility for protein function with structural robustness for enhanced durability.

Polymersomes, composed of self-assembling amphiphilic block copolymers, are well established as robust alternatives to liposomes and offer a much wider chemical parameter space.^4^ These polymeric vesicles have been shown to support functional reconstitution of some highly stable membrane proteins such as AqpZ^5,6^ and the bacterial channel forming proteins OmpF and Tsx.^7–9^ This is despite the larger hydrophobic thickness (2–3 times that of a lipid bilayer) and significantly higher viscosity of polymer membranes. The high conformational flexibility of block copolymers and the presence of shorter polymers that segregate around the protein are thought to be important factors in matching the hydrophobic core of the protein.^10^ However, the non-native structure, chemistry, mechanics and dynamics of polymer membranes are not appropriate for functional incorporation of a wider range of membrane proteins; indeed, we will see that the protein we study here is not functional in a purely polymeric vesicle.

More recently, hybrid vesicles (HVs) composed of a mixture of lipids and block copolymers have been shown to provide a compromise between the biocompatibility of liposomes and the stability and robustness of polymersomes.^11^ Thus far, this work has concentrated on materials characterisation of these hybrid vesicles and their application to drug delivery.^12–18^ Meier and colleagues have investigated the partitioning of membrane proteins OmpF and MloK1 within phase separated planar polymer–lipid films, however these studies did not demonstrate native folding or function of the proteins in these systems.^19,20^ Functional reconstitution of membrane proteins within hybrid lipid–diblock copolymer vesicle systems is yet to be investigated.

Here we report the functional reconstitution of the membrane protein cytochrome *bo*_3_ (cyt *bo*_3_) into hybrid vesicles composed of the phospholipid POPC and diblock copolymer poly(butadiene-*b*-ethylene oxide) (PBd_22_-*b*-PEO_14_) (Fig. 1). The material properties of PBd–PEO:POPC hybrid vesicles are well studied and are known to form homogeneous, well-mixed vesicles.^14–16^ Cyt *bo*_3_ is a membrane protein that belongs to the heme-copper oxidase superfamily of enzymes (Fig. 1a). It is a redox-driven proton pump that couples the reduction of molecular oxygen to the vectoral translocation of protons across a membrane.^21^ Cyt *bo*_3_ from *E. coli* is a ∼143 kDa protein complex comprised of 4 subunits; the crystal structure reveals conserved charged and polar residues within the trans-membrane domain of subunit I, which serve as binding sites for ubiquinol.^22^ Importantly, cyt *bo*_3_ has a quantitative functional assay that allows us to directly compare the protein activity in vesicles of different compositions from pure POPC liposomes to PBd–PEO polymersomes through a range of PHVs of different relative compositions. This is achieved by spectroscopic monitoring of the oxidation of reduced decylubiquinone (QH_2_ → Q) (Fig. 1b).

**Fig. 1:**
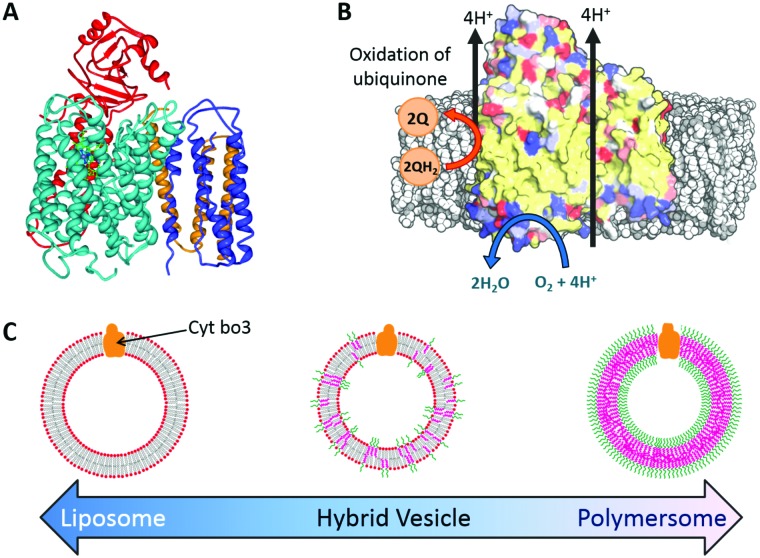
Reconstitution of cytochrome *bo*_3_ (cyt *bo*_3_) in hybrid vesicles. (A) Ribbon diagram of cyt *bo*_3_. (B) Schematic illustration of cyt *bo*_3_ reactions (C) schematic diagram of nanoscale proteo-phospholipid/block copolymer hybrid vesicles (PHVs).

Two methods of membrane protein reconstitution were investigated for effective incorporation of cyt *bo*_3_ into PHVs. In the first method, adapted from the proteoliposome literature,^23,24^ the membrane components were micellised in solution using the non-ionic detergent octyl β-d-glucopyranoside (OGP) and mixed with cyt *bo*_3_, which is stabilized in Triton X-100 detergent. The detergents are then removed using adsorbent bio-beads, triggering the self-assembly of PHVs. However, with the exception of proteoliposomes with an aggregate distribution centred at 200 nm, aggregate diameters for all membrane compositions containing block copolymer (25–100% composition) were centred around 15–17 nm (Fig. S1, ESI†). This is consistent with the dominance of polymer-rich micelles in these samples *via* this kinetic pathway of self-assembly. We hypothesise that the slow, viscous dynamics of block copolymers inhibits their rapid formation into vesicles during removal of micellising detergents. Therefore an alternative reconstitution method is required.

Our second reconstitution method, adapted from Geertsma *et al.*,^25^ consists of first forming hybrid vesicles by extrusion (100 nm filter size). These vesicles were then destabilised by gradual addition of small concentrations of detergent (Triton X-100) while measuring changes in the sample's optical density (OD_540 nm_). At the point where the detergent begins to break up the structural integrity of the vesicles, noted by a discontinuous change in the decreasing sample turbidity (see Fig. S2, ESI†), the vesicles were inoculated with cyt *bo*_3_ at a mixing ratio estimated to yield one reconstituted protein per 100 nm HV. Detergents were then removed using adsorbent biobeads. Dynamic light scattering (DLS) was again used to characterise sample size distributions before and after the addition of protein (Fig. 2). Vesicles were observed in all samples before and after protein reconstitution with composition-dependent diameter distributions centred between 75 nm (50% polymer) and 116 nm (100% polymer) for vesicles with reconstituted proteins (Table S1, ESI†). PHV formation was further confirmed by their ability to encapsulate a water soluble carboxyfluorescein dye and be efficiently separated from unencapsulated dye on a Sephadex G50 gel filtration column. We note that a population of micelles also coexist with hybrid vesicles composed of 50% and 75% polymer (before and after protein reconstitution) when prepared by this method, evident from the bimodal size distribution. This complicating factor for these two PHV compositions will be considered further in the subsequent analysis.

**Fig. 2:**
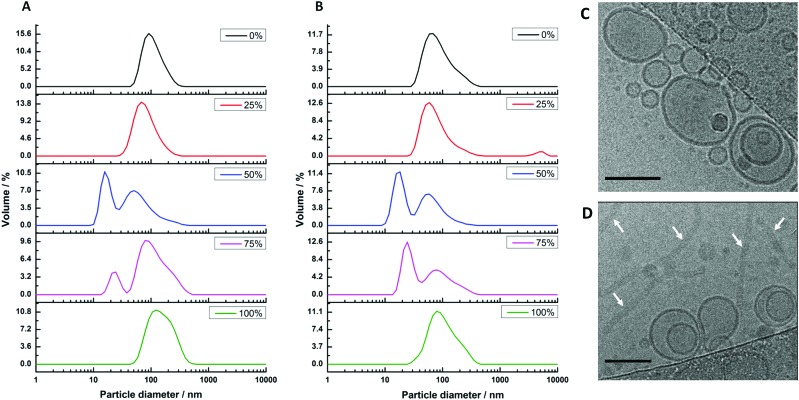
Size distributions of hybrid vesicles formed by method 2. The samples were prepared with varying block-copolymer mole fractions of 0% (lipid only), 25%, 50%, 75% and 100% (polymer only). (A) Sizes of extruded vesicles prior to cyt *bo*_3_ reconstitution; (B) sizes after cyt *bo*_3_ reconstitution. (C and D) Cryo-TEM images of (C) 25% PHVs and (D) 50% PHVs. Arrows in D point to worm-like micelles coexisting with the vesicles. Scale bars are 100 nm.

We further analysed 25% PHV and 50% PHV samples by cryo-TEM (Fig. 2C and D). Vesicles close to 100 nm in diameter are observed in both samples. Large worm-like micelles, multiple microns in length, were also seen in 50% PHV samples. Interestingly, interpretation of the DLS data had suggested a small 15–17 nm micelle population is present at this composition. This reinforces the care that needs to be taken when interpreting DLS data from highly non-spherical particles. In 50% PHV samples, it seems that scattering from internal undulation dynamics within large worm-like micelles is being measured, giving rise to fast relaxation modes consistent with the diffusion of 15–17 nm Brownian spheres.

The protein activities in the PHVs were compared by measuring the initial rates of decylubiquinone oxidation. Protein activity is seen to decrease with increasing polymer concentration in PHVs, with a fairly small reduction in activity from POPC liposomes to 50% PHVs, but more considerable reduction in activity for 75% PHVs and PBd–PEO polymersomes (Fig. 3). In fact, the 92% reduction in protein activity in polymersomes compares to the activity of cyt *bo*_3_ denatured by removal of its stabilising detergent. Therefore we conclude that cyt *bo*_3_ is not functionally reconstituted in PBd–PEO polymersomes due to the poor biocompatibility of its membrane. We conclude from DLS and initial protein activity data that our second reconstitution method facilitates the functional reconstitution of cyt *bo*_3_ within PHVs, where 25% PHVs provide the most unambiguous demonstration of this as only vesicle-sized particles are measured for this composition with no coexisting population of micelle-sized structures.

**Fig. 3:**
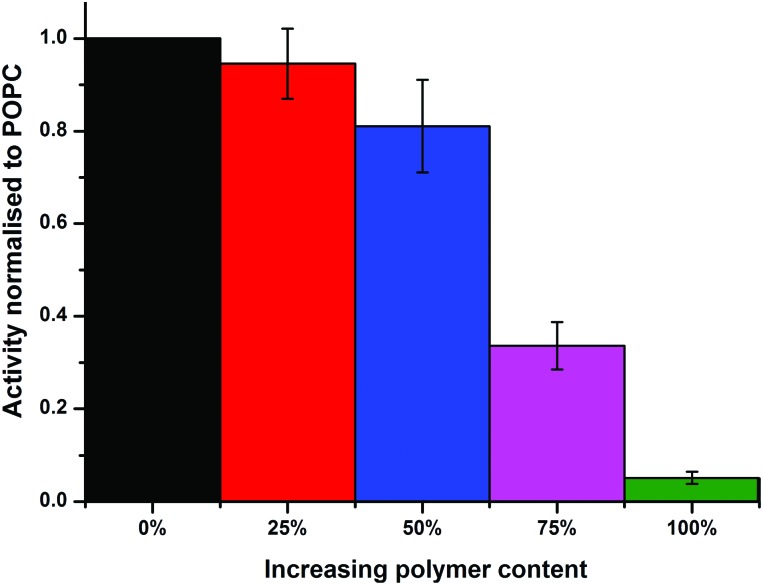
Initial protein activity is composition-dependent. Histogram of initial rates of decylubiquinone oxidation by cyt *bo*_3_ when reconstituted into PHVs of varying block copolymer content (0 to 100%). All values are normalised to the initial activity in POPC liposomes and error bars represent the standard error. Each measurement is the average of 7 independent PHV preparations, including 3 independently expressed samples of cyt *bo*_3_.

To test the long term durability of PHVs, we monitored the protein activity of samples over a period of six weeks. Samples were stored at 4 °C during this period. Fig. 4 shows the protein activity of PHVs normalised to their initial activity at day 0, following initial preparation. PBd–PEO polymersomes were not included in these studies because cyt *bo*_3_ did not functionally reconstitute within them. There is a clear trend that increasing the polymer content of PHVs significantly improves the protein's long term stability. Unambiguously enhancement of membrane protein durability is demonstrated in the 25% PHV systems, which exclusively consist of vesicles. These PHVs retain >40% of their initial activity after 41 days, compared to a 97% loss in activity in liposomes over the same period; proteoliposomes were almost completely inactive by day 28.

**Fig. 4:**
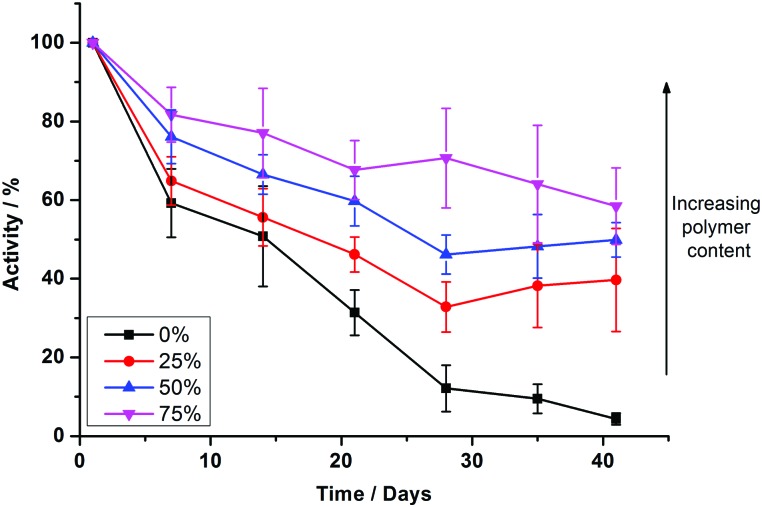
Increased copolymer composition prolongs the functional lifetime of reconstituted cyt *bo*_3_. Comparison of enzymatic activity of cyt *bo*_3_ in reconstituted PHVs prepared by method 2 over a period of 41 days. The protein activity is calculated with respect to the initial activity after preparation on day 0 and error bars represent the standard error. Each measurement is the average of 7 independent PHV preparations, including 3 independently expressed samples of cyt *bo*_3_.

An even greater degree of functional durability is demonstrated by the 50% and 75% PHV blends. These PHVs only dropped ∼40% in protein activity during the 6 week period. However, the interpretation of this data is complicated by the bimodal population of micelles and vesicles evident from the DLS data in Fig. 2; protein activity in the vesicles is combined with the activity of cyt *bo*_3_ that could be present in the micelle population. Due to the high initial protein activity measured in the 50% PHV samples (Fig. 3), combined with their enhanced functional durability (Fig. 4), we investigated these samples further by attempting to separate the mixed populations by size exclusion chromatography (SEC). We successfully separated vesicle-only fractions from vesicle and micelle containing fractions on a Sephadex G50 column, demonstrating the potential of SEC as a method of PHV purification in high polymer-content formulations (Fig. 5).

**Fig. 5:**
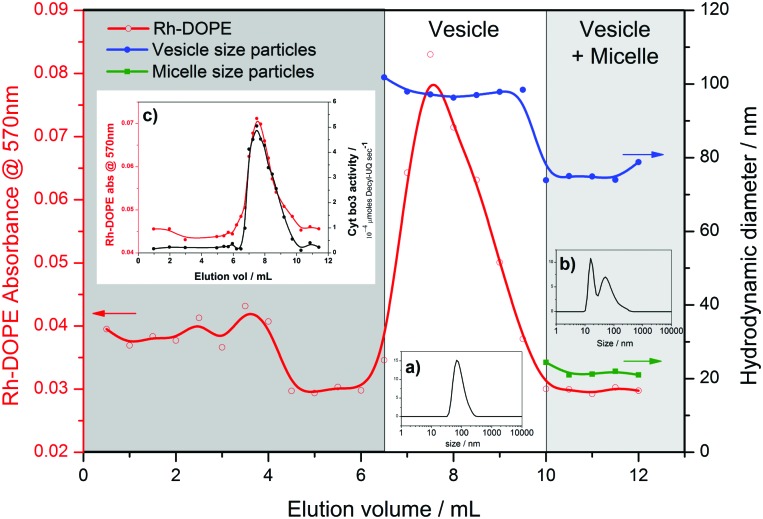
Purification of vesicles in 50% PHV samples. Fractions eluted from a Sephadex G50 column were analysed by adsorption spectroscopy (570 nm) and DLS. Size information is for the average of each peak in the distribution. Inset: size distributions for typical (a) vesicle only and (b) vesicle plus micelle fractions; (c) cyt *bo*_3_ activity of eluted fractions.

PHVs were labelled with 0.5 mol% Rhodamine-labelled DOPE lipid (Rh-DOPE) for the SEC separation experiments to help visually track them through the gravity column. Therefore this allowed us to quantify Rh-DOPE concentrations in each fraction by their adsorption at 570 nm (Fig. 5). Based upon an assumption that Rh-DOPE lipids distribute similarly to POPC lipids in PHVs, this suggests that lipids have a strong localisation with larger vesicles in 50% PHVs, whereas the lipids more evenly distribute across vesicle-only and vesicle plus micelle fractions for 75% PHVs (Fig. S3, ESI†). This, taken together with the correlation of Rh-DOPE adsorption with cyt *bo*_3_ activity of eluted fractions (Fig. 5c), is indicative that the functional durability of 50% PHV samples comes from the vesicles. To confirm this, we conducted long term stability assays on vesicles purified from 50% and 75% PHV samples (Fig. S4, ESI†). Purified 75% PHVs show a decay in activity within the error of the activity of unpurified samples in Fig. 4. Importantly, 50% PHVs show enhanced functional durability following column separation, indicative that the micelle population may have a small destabilising effect on protein function.

We have demonstrated that PHVs form robust and durable environments for the reconstitution of integral membrane proteins. The combined biocompatibility from lipids and the resilience from block copolymer membranes can support the high initial activity of cyt *bo*_3_ in PHVs (Fig. 3) and significantly extends the functional lifetime of the protein when compared to standard proteoliposomes (Fig. 4). This conclusion is most clearly demonstrated by 25% PHVs, where analysis of the results is not complicated by coexisting micelles within these samples. However the best combination of high initial activity and long term functional activity is found in the 50% PHV system, where interpretation of these findings is complicated by the coexistence of micelles within these samples. Some degree of separation of vesicles from micelle-containing fractions can be achieved by SEC using Sephadex G50 gravity columns, but future work will aim to enhance the degree of resolution in chromatographic purification of PHVs. Analysis of the separated fractions of PHVs demonstrates that the functional durability of cyt *bo*_3_ is provided by the vesicle population of the sample.

While the 50 : 50 POPC : PBd_22_-*b*-PEO_14_ PHV composition provided the best combination of high initial activity and slow loss in activity for cyt *bo*_3_, further optimisation of the PHV constituents (*e.g.* different polymers and/or lipids) and reconstitution protocols might further improve their long term stability. Our findings demonstrate that PHVs for reconstitution of membrane proteins in bionanotechnology can be a powerful tool to extend the range of proteins available through functional reconstitution into polymersomes and to increase the functional lifetime of these systems when compared to classical proteoliposomes.

PAB acknowledges funding from Marie Curie Career Integration Grant BioNanoMuTT (PCIG09-GA-2011-293643) and the University of Leeds. LJCJ and ML were funded by the European Research Council under the European Union's Seventh Framework Programme (FP/2007–2013)/ERC Grant Agreement no. 280518.
