# Supplementary material for: Durable proteo-hybrid vesicles for the extended functional lifetime of membrane proteins in bionanotechnology
Source: Chem Commun (Camb). 2016 Aug 16;52(73):11020–3. doi: 10.1039/c6cc04207d (PMC5048396; doi:10.1039/c6cc04207d)
Supplement: Supplementary file 1 [file CC-052-C6CC04207D-s001.pdf]

Electronic Supplementary Material (ESI) for ChemComm.
This journal is © The Royal Society of Chemistry 2016

Supplementary Information

Durable Proteo-Hybrid Vesicles for the Extended Functional Lifetime of Membrane Proteins in Bionanotechnology

Sanobar Khan,[a] Mengqiu Li,[b] Stephen P. Muench,[b] Lars J.C. Jeuken[b] and Paul A. Beales[a,*]

[a] School of Chemistry and Astbury Centre for Structural Molecular Biology, University of Leeds, Leeds, LS2 9JT, UK.

[b] School of Biomedical Sciences and Astbury Centre for Structural Molecular Biology, University of Leeds, Leeds, LS2 9JT, UK.

* E-mail: p.a.beales@leeds.ac.uk.

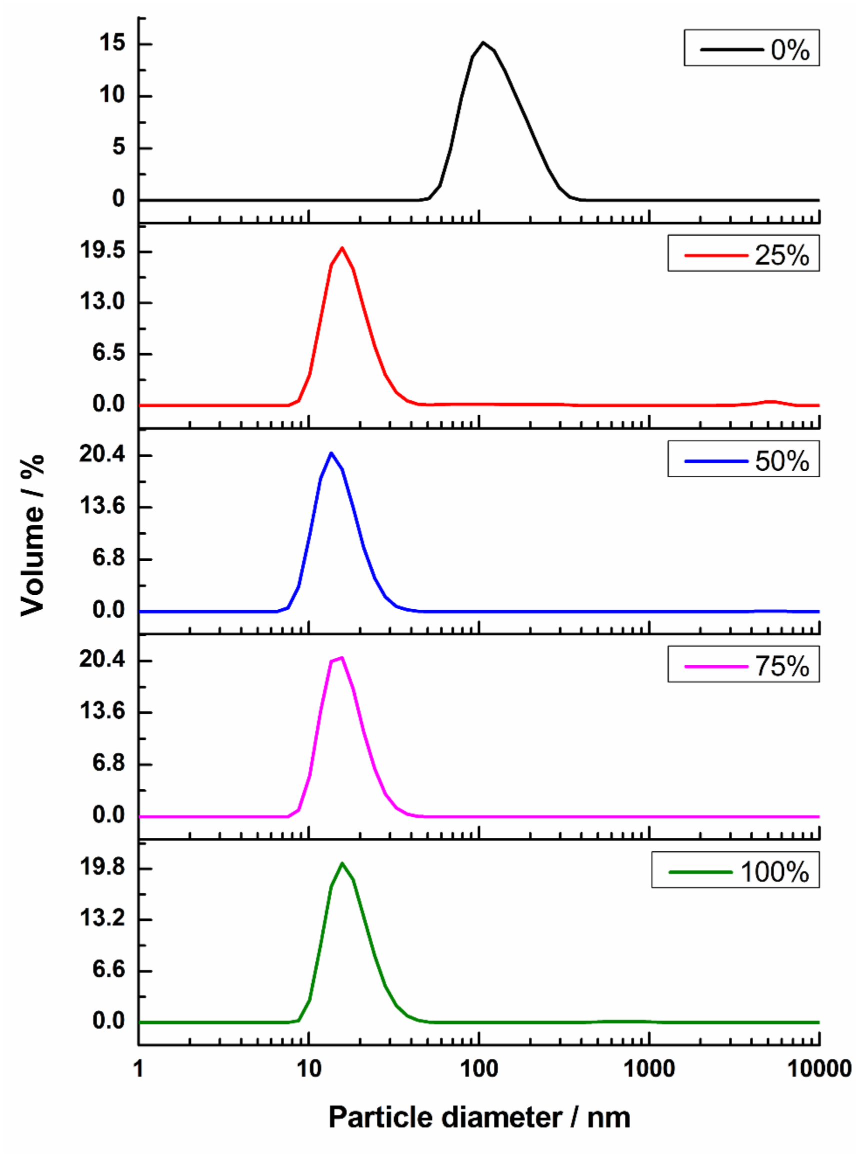

Figure S1. Aggregate size distributions by reconstitution method 1. Hydrodynamic diameter distributions for samples containing different mol% block copolymer after detergent extraction using Bio-Beads, as described in Method 1.

Polymer mol%	Z-Average (d/nm)	PdI		Size (d/nm)	% Volume	St Dev (d/nm)
0%	166.40	0.141	Peak 1	196.4	100	100.8
25%	57.70	0.632	Peak 1	147.5	1.7	89.36
			Peak 2	17.38	96.7	5.34
			Peak 3	4839	1.6	944.7
50%	30.17	0.467	Peak 1	15.28	99.5	4.604
			Peak 2	200.2	0.3	98.28
			Peak 3	5047	0.1	828.3
75%	29.55	0.442	Peak 1	16.54	99.7	4.829
			Peak 2	281.4	0.3	123
100%	26.26	0.361	Peak 1	17.91	99.5	5.533
			Peak 2	718.6	0.5	312.9

Table S1. Table summarising DLS results from data in Fig. S1. Z-average particle diameter (d/nm), Polydispersity Index (PdI), Size peaks from the diameter distribution profile and corresponding % Volume for samples containing different polymer:lipid ratios (expressed in block copolymer mol%).

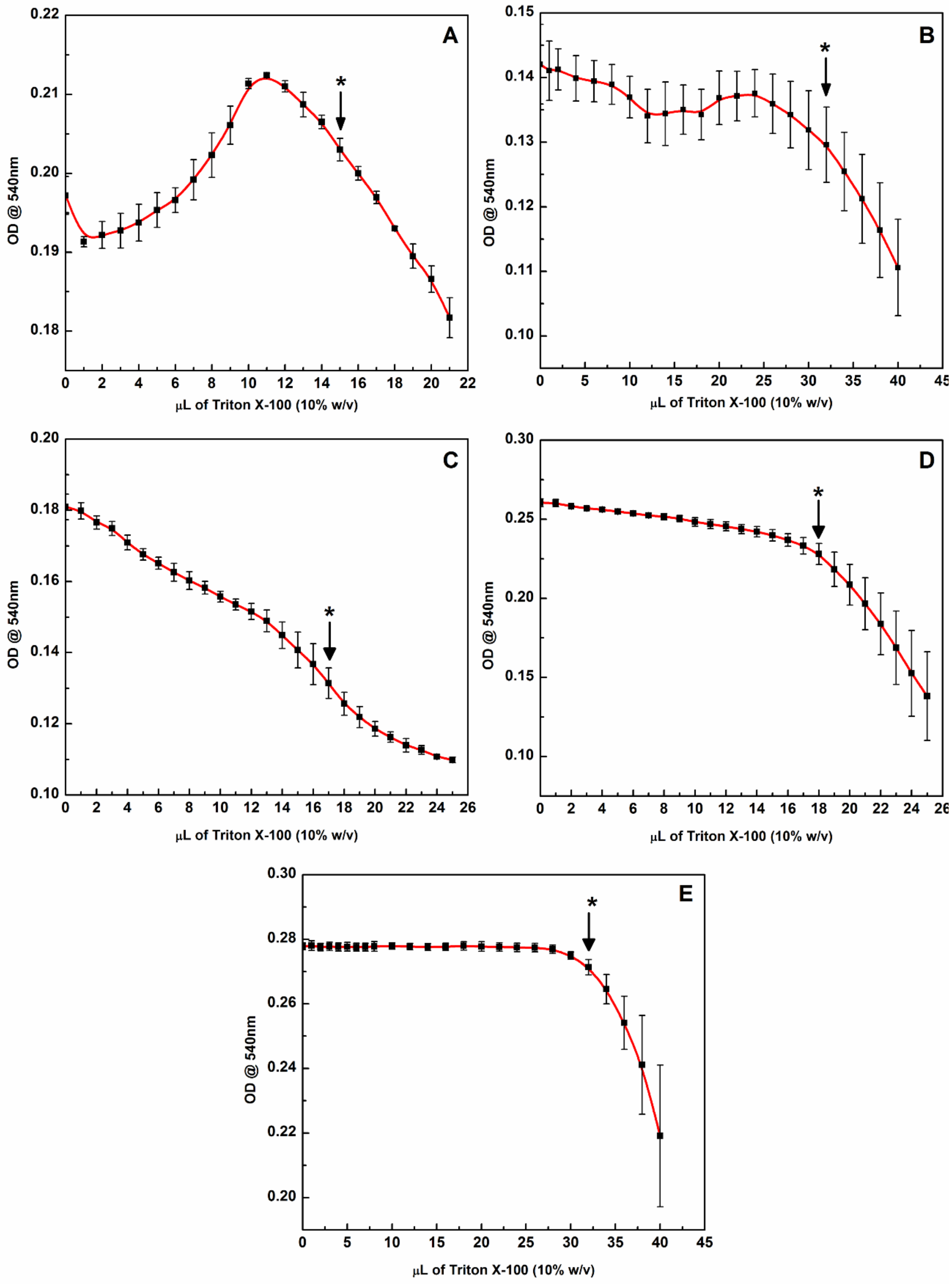

Figure S2. Detergent destabilisation profiles for; A) POPC liposomes (0% polymer), *=15µL; B) HV containing 25% polymer, *=35µL; C) HV containing 50% polymer, *=16µL; D) HV containing 75% polymer, *=17µL; E) PBD$_{22}$-b-PEO$_{14}$ polymersomes (100% polymer), *=30µL, prepared via the extrusion method described in Method 2. Plot shows change in OD$_{540nm}$ upon the addition of Triton-X100 (10% w/v). The destabilisation point chosen for reconstitution is indicated on the graphs (*).

Hybrid Vesicles prepared via extrusion method						
Polymer mol%	Z-Average (d.nm)	PDI		Size (d.nm)	% Volume	St Dev (d.nm)
0%	119.80	0.093	Peak 1	114.2	100	45.47
25%	95.05	0.165	Peak 2	86.55	100	40.59
50%	94.25	0.233	Peak 1	75.43	57.9	53.37
			Peak 2	17.9	42.1	4.464
75%	125.60	0.19	Peak 1	130	85.3	75.42
			Peak 2	23.96	14.7	4.779
100%	151.20	0.117	Peak 1	162.9	100	77.39
After vesicle destabilisation and detergent extraction						
Polymer mol%	Z-Average (d.nm)	PDI		Size (d.nm)	% Volume	St Dev (d.nm)
0%	109.90	0.167	Peak 1	96.54	100	58.65
25%	98.47	0.219	Peak 1	85.08	96.5	55.27
			Peak 2	4866	3.5	913.3
50%	85.88	0.229	Peak 1	75.76	49.9	42.79
			Peak 2	18.78	50.1	5.075
75%	112.60	0.192	Peak 1	110.3	49.6	63.07
			Peak 2	26.5	50.4	6.834
100%	123.30	0.15	Peak 1	115.2	100	66.07

Table S2: Table showing DLS results for pHVs prepared via method 2 corresponding to the size distributions graphed in Fig. 2. The data shows Z-average particle diameter (d/nm), Polydispersity Index (PdI), Size peaks form the particle size distribution profile and corresponding % Volume for samples containing different polymer:lipid ratios (expressed in polymer mol%).

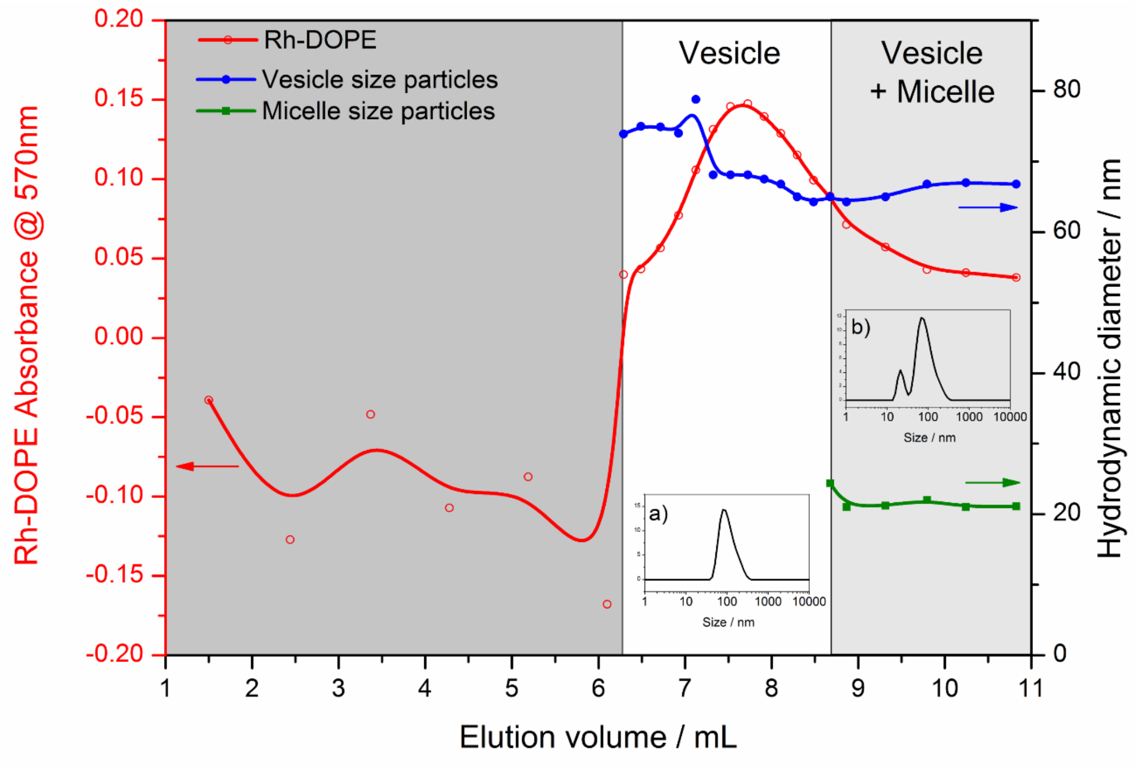

Figure S3: Purification of vesicles in 75% PHV samples. Fractions eluted from a Sephadex G50 column were analysed by adsorption spectroscopy (570 nm) and DLS. Size information is for the average of each peak in the distribution. Inset DLS size distributions are shown for typical (a) vesicle only and (b) vesicle plus micelle samples. Note that the boundaries between vesicle only and vesicle plus micelle elution volumes is determined analytically from the DLS of eluted fractions; the exact range of elution volumes seen here differs from the 50% PHV samples in Fig. 5 likely due to differences in the size of vesicles at these compositions (see table S2) and variability in these separation experiments that are run on a benchtop gravity column.

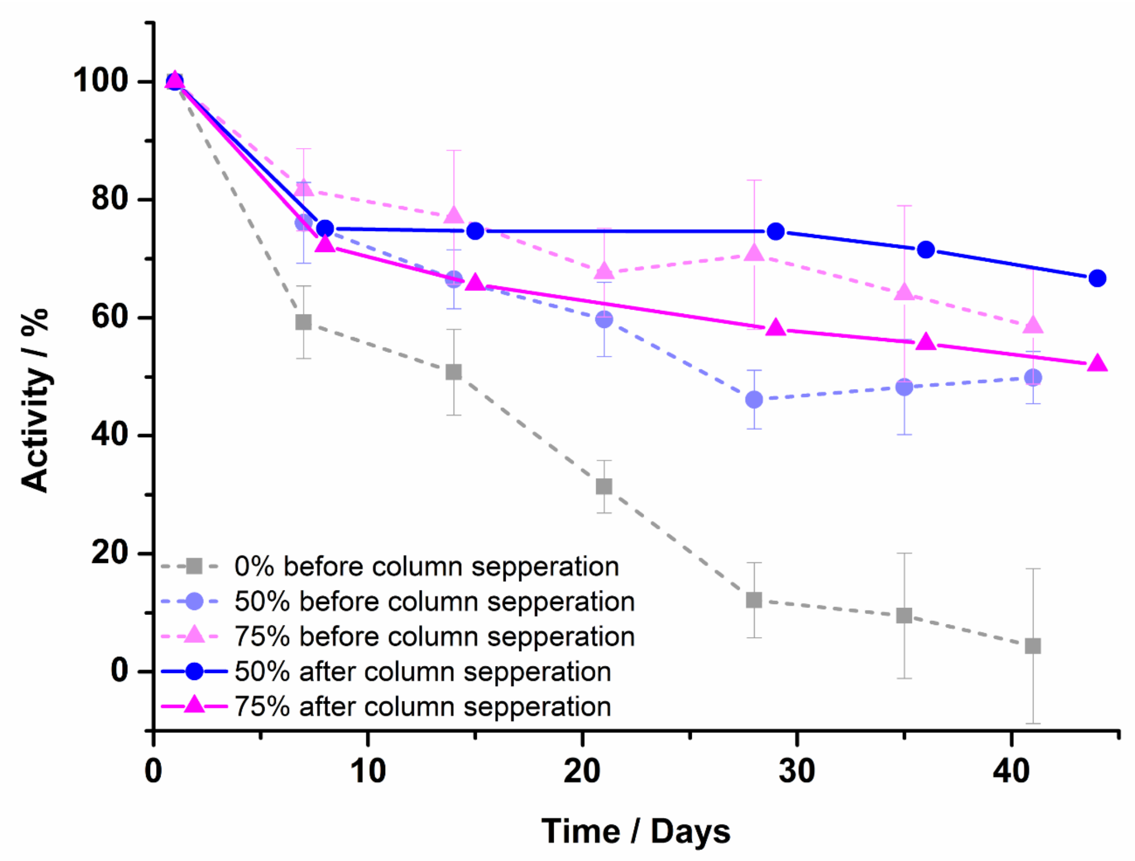

Figure S4: Enzymatic activity of cyt bo3 over time, before and after purification of vesicles in 50% and 75% PHV samples. Comparison of enzymatic activity of cyt bo3 in reconstituted PHVs prepared by method 2 and separated on a Sephadex G50 column, over a period of 44 days. The protein activity is calculated with respect to the initial activity after preparation on day 0 and error bars represent the standard error. Each measurement is the average of 2 independent PHV preparations.

Methods

Cytochrome bo3 Expression and Purification

Wild-type cytochrome *bo*$_3$ (cyt *bo*$_3$) was purified from GO105/pJRhisA and purified similarly to methods previously reported.[1]

Reconstitution Method 1

The first method was adapted from Galian, et al. [2] and Mager, et al. [3]. Solutions of 1-palmitoyl-2-oleoyl-sn-glycero-3-phosphocholine (POPC; Avanti Polar Lipids, CAS no. 26853-31-6) and block-copolymer, Poly(butadiene-b-ethylene oxide) (PDB_{22}-b-PEO_{14}; Polymer Source, P9089-BdEO), 32.89 mM in chloroform, were mixed in glass vials to five different compositions to give block-copolymer mole fractions of 0% (lipid only), 25%, 50%, 75% and 100% (polymer only). These where then dried under a vacuumed desiccator overnight to give lipid / polymer films at the bottom of the glass vials. The lipid / block-copolymer films were rehydrated with 1 mL of buffer containing 20 mM 4-(2-hydroxyethyl)-1-piperazineethanesulfonic acid (HEPES), 10 mM sodium chloride and 55 mM Octyl β-D-glucopyranoside (OGP; Sigma-Aldrich, CAS no. 29836-26-8), pH 7.4, and vortexed until the lipid / block-copolymer film was completely solubilised in the buffer. To 450 µL of the solubilised lipid / block-copolymer, 5µg of purified cyt *bo*$_3$ was added and allowed to inoculate for 15 min at 4 °C.

The detergent removal was carried out by four successive cycles with Bio-Beads (Bio-Beads SM-2 adsorbents, Bio-Rad) while the samples were incubated on a rocker at 4 °C. Cycle 1 = 25 mg Bio-Beads, incubate for 30min; Cycle 2 = 25 mg Bio-Beads, incubate for 1 h; Cycle 3 = 50 mg Bio-Beads, incubate for 1 h; Cycle 4 = 50 mg Bio-Beads, incubate for 2 h. The Bio-Beads were removed at the end of each cycle and fresh Bio-Beads were introduced.

Reconstitution Method 2

The second method was adapted from Geertsma, E.R. et al.[4] Lipid / block-copolymer films were prepared as described in method 1. Vesicles were formed by adding buffer (20 mM HEPES, 10 mM NaCl, pH 7.4), followed by incubation at 50 °C for 5 min and vortex for 1 min, then incubate for further 5 min and vortex again. The resultant suspension was put through five freeze-thaw-vortex cycles and subsequently extruded 11 times through a 100 nm pore size polycarbonate membrane filter using an Avanti Mini-Extruder to form nanovesicles.

The prepared 1 mL of vesicles are destabilised by titrating with 1 µL aliquots additions of 10% (w/v) Triton X-100 (Sigma-Aldrich, CAS no. 9002-93-1) while observing the optical density at 540 nm (Figure 6). To 500 µL of destabilised vesicles, 5 µg of purified cyt *bo*$_3$ was added and allowed to inoculate for 15 min at 4 °C. The detergent removal was carried out by four successive cycles with Bio-Beads (Bio-Beads SM-2 adsorbents, Bio-Rad) while the samples were incubated on a rocker at 4 °C. (Cycle1 = 20 mg Bio-Beads, incubate for 30 min; Cycle 2 = 20 mg Bio-Beads, incubate for 1 h; Cycle 3 = 20 mg Bio-Beads, incubate for 6 h; Cycle 4 = 20 mg Bio-Beads, incubate for 6 h).

PHV Characterisation

To verify the formation of vesicular structures with both Method 1 and Method 2, the samples were analysed using dynamic light scattering (DLS) as well as using Carboxyfluorescein encapsulation and leakage experiments. DLS experiments were performed using a Malvern Zetasizer Nano ZS: the sample was measured at a fixed 173° scattering angle. Three measurements for each sample were performed at 25 °C and the vesicle size was reported as the average of the three measurements.

For electron microscopy analysis Lacey grids (Agar Scientific) were glow discharged for 10 seconds using an EasiGlow system. Following this 3.0 µl of the PHV sample (6.5 mM amphiphile (lipid + block copolymer) concentration) was applied to the grid which was subsequently blotted for 4 s at blot force 2, using a Vitrobot mark IV. Grids were imaged on an FEI F20 microscope working at 200 KV and operating in low dose mode. Images were collected on a Gatan 4K x 4K CCD detector at a range of magnifications.

Carboxyfluorescein (CF) encapsulation: For Method 1 the solubilisation buffer was switched to 20 mM HEPES, 10 mM NaCl, 55 mM OGP and 125 mM 5(6)-Carboxyfluorescein (Sigma-Aldrich, cas 72088-94-9), pH 7.4. For Method 2 the hydration buffer was switched to 20 mM HEPES, 10 mM NaCl, 125 mM CF, pH 7.4.

Unencapsulated CF was removed via gel filtration using a Sephadex G50 medium column, eluting with HEPES buffer without CF. To destabilise the vesicles and release encapsulated CF, 50µL of 10% Triton X-100 (w/v) was added to the sample and the fluorescence emission was recorded as counts per second (cps). The increase in fluorescence output was calculated by subtracting the background emission from the fluorescence emission recorded after the addition of Triton X-100.

Protein activity assay

The enzymatic activity assay was similar to that outlined in Rumbley, et al. [5]. First the substrate, Decylubiquinone (acquired form Sigma-Aldrich, CAS No: 55486-00-5), was solubilised in absolute ethanol. The concentration of the prepared decylubiquinone solution was confirmed spectroscopically at 275 nm (ε = 19 $mM^{-1}cm^{-1}$ in absolute ethanol) to be 1.695 mM. For the assay, decylubiquinone (DUQ) was reduced to decylubiquinol (DUQH2) using sodium borohydride crystals in a method similar to that described in Trounce, I.A. et al.[6] To remove excess hydride ions (H^-) remaining in the solution, 0.1 M HCl was added (1 µL per 10 µL of DUQ solution). For the assay, 70 µL of prepared PHV, and 20 µL of 1.695 mM $DUQH_2$ was diluted in HEPES buffer (40 mM HEPES, 20 mM NaCl, pH 7.4) to a final volume of 700 µL and the oxidation of $DUQH_2$ was monitored at 275 nm (ε = 12.25 $mM^{-1}cm^{-1}$ in aqueous solution [7]) for 5 min.

The activity of cyt bo_3 is calculated by converting the absorbance reading at 275 nm into µmoles of Decylubiquinone (using ε = 12250 M^{-1} cm^{-1}) and plotted against time. The initial slope of the curve in a plot of µmoles of Decylubiquinone against time (gradient of the initial 20 seconds) is calculated and converted to represent rate of Decylubiquinone turnover per ml of prepared sample. The resulting value thus represents the enzymatic activity of cyt bo_3. The raw data had some noise due to light scattering from the vesicles and this contributes to the overall error in the calculated enzymatic activity.

Chromatographic separation of PHVs

PHV samples were run on a Sephadex G50 gravity column. 0.5 mol% 1,2-dioleoyl-sn-glycero-3-phosphoethanolamine-N-(lissamine rhodamine B sulfonyl) (ammonium salt) (Rh-DOPE; Avanti Polar Lipids; CAS no. 384833-00-5) was included in the membrane of these PHVs during formation so they could be visually tracked on the column. 0.5 ml eluted fractions were collected and quantified by UV-vis adsorption spectroscopy at 570 nm (to measure Rh-DOPE content) and dynamic light scattering for particle size distributions. These fractions were further analysed for their protein activity as described above.

References

[1] J. N. Rumbley, E. F. Nickels, R. B. Gennis, *Biochim. Biophys. Acta* **1997**, *1340*, 131-142.

[2] C. Galian, F. Manon, M. Dezi, C. Torres, C. Ebel, D. Levy, J. M. Jault, *PloS one* **2011**, *6*, e19677.

[3] T. Mager, A. Rimon, E. Padan, K. Fendler, *J. Biol. Chem.* **2011**, *286*, 23570-23581.

[4] E. R. Geertsma, N. A. B. Nik Mahmood, G. K. Schuurman-Wolters, B. Poolman, *Nat. Protocols* **2008**, *3*, 256-266.

[5] J. N. Rumbley, E. Furlong Nickels, R. B. Gennis, *Biochim. Biophys. Acta* **1997**, *1340*, 131-142.

[6] I. A. Trounce, Y. L. Kim, A. S. Jun, D. C. Wallace, in *Methods Enzymol., Vol. 264*, Academic Press, **1996**, pp. 484-509.

[7] F. L. Crane, R. Barr, in *Methods Enzymol., Vol. 18, Part C* (Eds.: B. M. Donald, D. W. Lemuel), Academic Press, **1971**, pp. 137-165.
